# Central extracorporeal membrane oxygenation for treatment of reperfusion oedema following pulmonary thromboendarterectomy: a case report

**DOI:** 10.1186/s13019-016-0476-1

**Published:** 2016-05-04

**Authors:** Alexander Edemskiy, Mikhail Chernyavskiy, Alexandra Tarkova, Alexander Chernyavskiy

**Affiliations:** Department of Aorta and Coronary Arteries Surgery, Novosibirsk Research Institute of Circulation Pathology n.a. Academician E.N. Meshalkin, 15 Rechkunovskaya street, Novosibirsk, 630055 Russia

**Keywords:** Сhronic thromboembolic pulmonary hypertension, Pulmonary thromboendarterectomy, Lung reperfusion injury, Extracorporeal membrane oxygenation

## Abstract

One of the most severe and frequent complication of pulmonary thromboendarterectomy is reperfusion pulmonary oedema. The only effective treatment for this complication is extracorporeal membrane oxygenation. A case of successful treatment of reperfusion pulmonary oedema with prolonged veno-arterial extracorporeal membrane oxygenation complicated by several episodes of bleeding and surgical site infection is presented.

## Background

Pulmonary thromboendarterectomy (PTE) is currently the gold standard treatment for chronic thromboembolic pulmonary hypertension (CTEPH) [1]. Intraoperative pulmonary haemorrhage, lung reperfusion oedema and the consequent development of right heart failure in the early postoperative period are the most severe and often fatal complications of this procedure. Often, the only option for treatment of these serious complications is the use of extracorporeal membrane oxygenation (ECMO) during the perioperative period [2, 3]. We present a clinical case of the successful use of ECMO after PTE.

## Case presentation

A 31-year-old woman was hospitalised with a diagnosis of CTEPH, relapsing pulmonary thromboembolism and New York Heart Association functional class III heart failure. She had noted in 2013 the appearance of dyspnea that first occurred during light exercise and subsequently progressed. In August 2013, she was diagnosed with relapsing pulmonary thromboembolism. Echocardiography revealed a high pulmonary artery (PA) pressure of 54 mm Hg, dilated right heart chambers, and second-degree tricuspid insufficiency; multi-spectral computed tomography (CT) revealed signs of thrombosis in the segmental branches of both PAs. However, the patient was not referred to a specialty surgical centre for assessment of her operability but was instead prescribed warfarin as an anticoagulant treatment. After a short period of stabilisation of her condition, the patient was hospitalised at her local healthcare facility for angiopulmonography and right heart catheterisation; her PA pressure was 110/34/59 mmHg, and chronic massive emboli of both branches of the PA were confirmed. The specialists at the referral centre reviewed her medical data and recommended PTE.

**Fig. 1:**
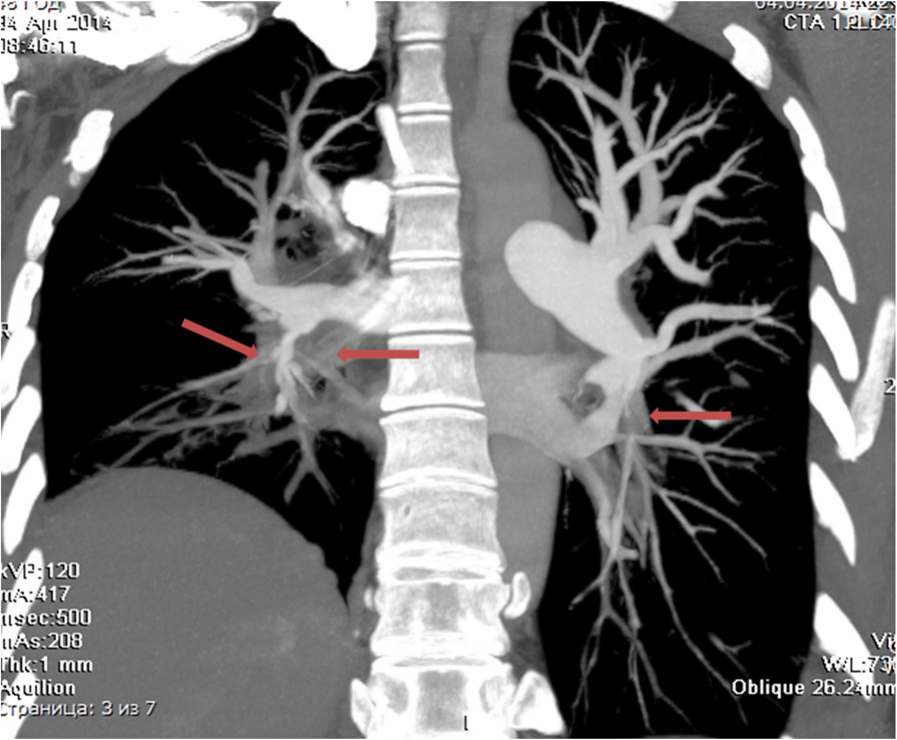
Preoperative multi-spectral CT angiography of the pulmonary artery; Figure demonstrates proximal lesion of pulmonary arteries, that was indicated with red indicator

The patient complained of dyspnoea after walking 100 m at her usual speed or while climbing one flight of stairs. The result of a 6-min walk test was 175 m. Echocardiography revealed dilated right heart chambers; a 20 % fractional area change (FAC) of the right ventricle, a PA pressure of 110 mmHg, and degree 2–3 of tricuspid regurgitation with a moderate regurgitate volume. Preoperative multi-spectral CT angiography of the PA (Fig. 1) revealed occlusive and stenosing parietal thrombi in the lumen of the segmental and subsegmental arteries of both lungs. Angiopulmonography revealed occlusion of the right upper lobar artery and left lower lobe segmental arteries. Right heart catheterisation revealed a PA pressure of 94/49/64 mmHg, PA wedge pressure of 15 mmHg, cardiac output of 3.0 L/min and pulmonary vascular resistance of 1306 dyn/s/cm^–5^. Considering the proximal location of the lesions in the branches of the PA, PTE was performed. A permanent vena cava filter was implanted.

**Fig. 2:**
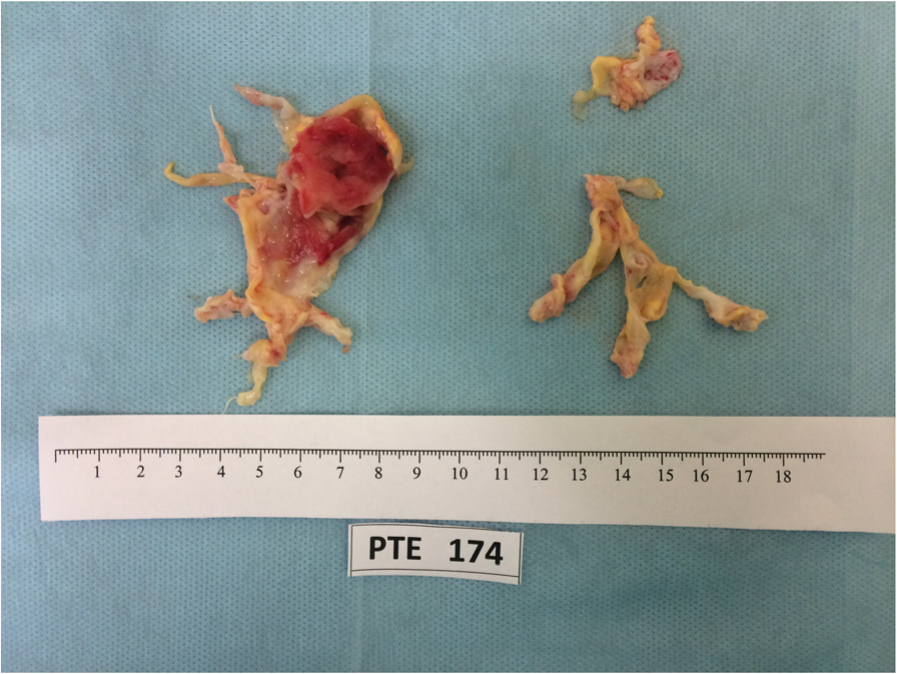
Removed organized thrombi and clots; Figure demonstrates organized thrombi and clots, that was removed from pulmonary artery during pulmonary thromboendarterectomy procedure

On 3 July 2014, PTE was carried out. The surgery was performed with deep hypothermia (18°С) and circulatory arrest using a standard procedure developed under the direction of S. Jamieson (University of California, San Diego, CA, USA). The thrombi that were removed (level 2 according to the University of California, San Diego classification) are shown in Fig. 2. The total duration of circulatory arrest was 39 min. After completely rewarming the patient and weaning from cardiopulmonary bypass, the PA pressure increased to 80/50 mmHg (simultaneously directly measured systemic blood pressure in the femoral artery was 70/50 mmHg), and her arterial blood oxygen saturation decreased. Considering the pulmonary injury induced by reperfusion as well as the left ventricular failure, we performed veno-arterial ECMO using the femoral vein-ascending aorta approach. The arterial line of the cardiopulmonary bypass circulation system was connected to the arterial line of the ECMO system. The venous line of the ECMO system was connected by puncture of the left femoral vein. The volume rate of blood perfusion was 4 L/min. The duration of artificial blood circulation was 292 min. The duration of occlusion of the aorta was 112 min.

**Fig. 3:**
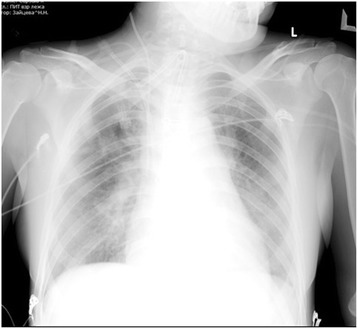
Chest X-ray: reperfusion pulmonary oedema after pulmonary thromboendarterectomy; Figure demonstrates the development of reperfusion pulmonary injury immediately after pulmonary thromboendarterectomy procedure

Echocardiographic examination carried out during the early postoperative period revealed diffuse myocardial hypokinesis of the right and the left ventricles, a left ventricular end-diastolic volume (LVEDV) of 65 mL, a right ventricular end-diastolic volume (RVEDV) of 26 mL, an ejection fraction (EF) of left ventricles of <20 % and an FAC of the right ventricle of <20 %. Notwithstanding the ECMO procedure, the patient had residual pulmonary hypertension (PA pressure, 68/24/39 mmHg; pulmonary vascular resistance, 615 dyn/s/cm^−5^). After the initiation of inhalation therapy with Iloprost (2 mL/ampoule, 10 mcg/mL every 3 h), the PA pressure decreased to 45/19/28 mmHg, and the pulmonary vascular resistance decreased to 216 dyn/s/cm^−5^. Considering the development of lung reperfusion injury (Fig. 3), artificial pulmonary ventilation and ECMO were continued notwithstanding the normalisation of the PA pressure.

**Fig. 4:**
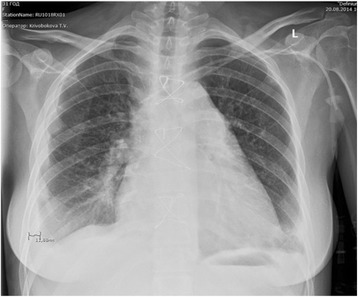
Chest X-ray: dynamics of reperfusion pulmonary oedema after pulmonary thromboendarterectomy, the 18^th^ day of ECMO; Figure demonstrates dynamics of reperfusion pulmonary oedema

On postoperative day 7, resternotomy and haemostasis were carried out in view of the excessive amount of haemorrhagic discharge from the drains and the presence of cardiac tamponade confirmed by echocardiography. The bleeding originated from the purse-string suture of the inflow aortic cannula. The patient thereafter exhibited positive changes on a background of 100 % ECMO: both ventricles demonstrated improvement in their myocardial contractility (LVEDV, 39 mL; left ventricular EF, 46 %; RVEDV, 25 mL; right ventricular FAC, 30 %). On postoperative day 12, the patient was haemodynamically stable, required no inotropic support, and had normal arterial and venous blood gas analysis results; additionally, the volume rate of ECMO blood perfusion had decreased to 50 %. On postoperative day 18, considering the patient’s risk of irreversible blood coagulation impairment and her satisfactory cardiac function, we removed the ECMO system with a tentative plan to discontinue its operation. X-ray examinations demonstrated a significant decrease in the severity of the lung reperfusion injury (Fig. 4).

**Fig. 5:**
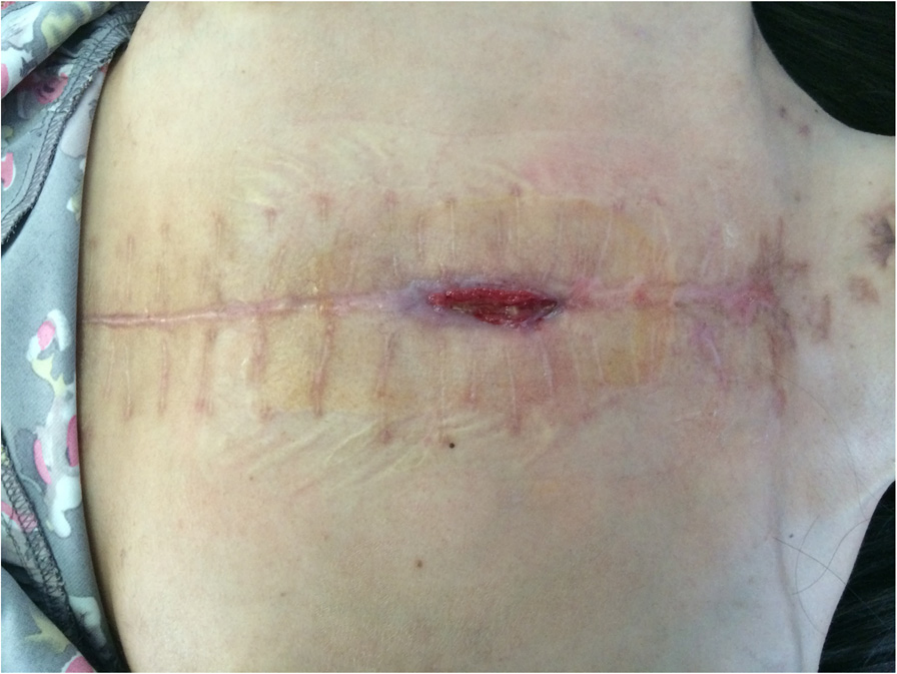
Secondary intention of operative wound, 62^th^ postoperative day; Figure demonstrates result of wound healing by secondary intension

The patient was brought to the operating room, where resternotomy was carried out. Next, after stopping the ECMO apparatus, the patient was decannulated. The day after ECMO removal, the patient exhibited manifestations of sepsis (i.e., positive blood culture, fever, and increasingly severe multi-organ failure as evidenced by oliguria and hyperbilirubinaemia), and antibacterial therapy based on the obtained bacterial sensitivity was administered. The patient thereafter showed positive changes: signs of consciousness appeared, and artificial pulmonary ventilation was changed to assisted ventilation. However, she still had a fever and leucocytosis with a left shift (i.e., high band neutrophil count). On postoperative day 32, during the bandaging, a 15-mm-long wound was noted in the middle third of the sutured surgical incision over the sternum; this wound produced a copious amount of serous discharge, and the sternum was unstable at its base. Considering the patient’s severe clinical condition and high risk associated with repeated general anaesthesia, we abstained from continuous-flow irrigation and draining of the anterior mediastinum. The wound was connected to a Suprasorb vacuum drainage system (Lohmann & Rauscher, Neuwied, Germany). On postoperative day 41, the patient was transferred to the somatic department in an alert conscious state, breathing without assistance and showing stable haemodynamic parameters. On postoperative day 46, the Suprasorb drainage system was removed. In view of the favourable course of vacuum therapy and good wound healing, the wound was left to heal by second intention (Fig. 5).

**Fig. 6:**
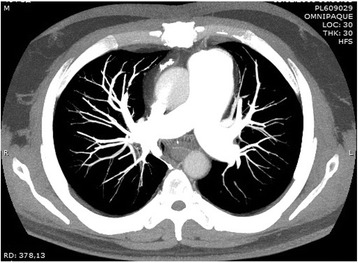
Multi-spectral CT angiography of the pulmonary artery 8 month after discharge; Figure demonstrates follow-up CT-angiography investigation

On postoperative day 67, after confirming positive changes on follow-up multi-spectral CT angiography (i.e., decreased diameter of the PA and presence of contrast agent in the previously occluded segmental and subsegmental branches in both lungs), the patient was discharged home with instructions to undergo follow-up by a cardiologist in her local healthcare facility. The result of the 6-min walk test was 282 m. Eight months after the operation the patient was invited for a follow-up evaluation. The result of the 6-min walk test was 350 m. Follow-up multi-spectral CT angiography of the PA (Fig. 6) failed to identify any new occlusive or stenotic lesions. Right heart catheterisation revealed a PA pressure of 46/20/29 mmHg, PA wedge pressure of 14 mmHg, cardiac output of 3.2 L/min, and pulmonary vascular resistance of 375 dyn/s/cm^−5^.

## Conclusions

Lung reperfusion oedema is one of the most severe complications of PTE. No clear prognostic criteria for the development of this postoperative complication have been established. It is impossible to clearly explain the fact that some patients do not develop clinically significant pulmonary oedema after quite extensive PTE, whereas other patients develop reperfusion injury after a less extensive procedure. However, it can be supposed that patients with a long history of CTEPH, a haemodynamic disorder (with pulmonary vascular resistance of >1000 dyn/s/cm^−5^), and decompensated right ventricular failure (with ascites, hepatomegaly, and lower extremity oedema) have a higher risk of reperfusion injury. At present, ECMO is the method of choice for treatment of reperfusion pulmonary oedema [4, 5].

We have presented a clinical case of successful 18-day veno-arterial ECMO using the femoral vein-ascending aorta approach after PTE. The most notable aspects of this case are as follows. First, the use of ECMO made it possible to wean the patient from cardiopulmonary bypass. Second, extracorporeal blood circulation resulted in a decrease in the pulmonary circulation that reduced the expression of reperfusion pulmonary injury. Third, the use of ECMO made it possible to reduce the manifestations of right ventricular failure, thus improving blood circulation in the whole body. Such prolonged use of ECMO can be complicated by haemostatic disorders in the form of disseminated intravascular coagulation syndrome. In our case use of Iloprost resulted in reduction or residual pulmonary hypertension. However, further research and observations on the use of this drug in such patients are requested. Additionally, the central ECMO cannulation approach (in our case, the arterial blood return was ensured through the ascending aorta) is associated with a high risk of mediastinitis (our patient had this complication). In our case, the use of vacuum therapy allowed us to stabilise the process, ensure second-intention wound healing, and avoid reoperation for re-osteosynthesis and continuous-flow irrigation and draining. During the long-term follow-up evaluation (8 months after the operation), the patient noted clear positive changes in her well-being that were confirmed by the results of the 6-min walk test and follow-up multi-spectral CT angiography and right heart catheterisation.

### Consent

Written informed consent was obtained from the patient for publication of this Case report and any accompanying images. A copy of the written consent is available for review by the Editor-in-Chief of this journal.

## Abbreviations

ACT: activated clothing time
CT: computed tomography
CTEPH: chronic thromboembolic pulmonary hypertension
ECMO: extracorporeal membrane oxygenation
EF: ejection fraction
FAC: fractional area change
LVEDV: left ventricle end-diastolic volume
NYHA: New-York heart association
PA: pulmonary artery
PTE: pulmonary thromboendarterectomy
RVEDV: right ventricle end-diastolic volume
UCSD: University California San-Diego

## References

[1] Jamieson SW, Kapelanski DP, Sakakibara N, Manecke GR, Thistlethwaite PA, Kerr KM (2003). Pulmonary endarterectomy: experience and lessons learned in 1,500 cases. Ann Thorac Surg.

[2] Pretorius V, Alayadhi W, Modry D (2009). Extracorporeal life support for the control of life-threatening pulmonary hemorrhage. Ann Thorac Surg.

[3] Berman M, Tsui S, Vuylsteke A, Snell A, Colah S, Latimer R (2008). Successful extracorporeal membrane oxygenation support after pulmonary thromboendarterectomy. Ann Thorac Surg.

[4] Faggian G, Onorati F, Chiominto B (2011). Veno-venous extracorporeal membrane oxygenation as a bridge to and support for pulmonary thromboendarterectomy in misdiagnosed chronic thromboembolic pulmonary hypertension. Artif Organs.

[5] Kramm T, Eberle B, Krummenauer F (2003). Inhaled iloprost in patients with chronic thromboembolic pulmonary hypertension: effects before and after pulmonary thromboendarterectomy. Ann Thorac Surg.

